# Single-Stage Craniectomy and Cranioplasty for Multilobular Osteochondrosarcoma Managed with a Custom Additive Manufactured Titanium Plate in a Dog

**DOI:** 10.1155/2019/6383591

**Published:** 2019-12-02

**Authors:** Galina M. Hayes, Elena A. Demeter, Eunju Choi, Michelle Oblak

**Affiliations:** ^1^Department of Clinical Studies, Section of Small Animal Surgery, Cornell University College of Veterinary Medicine, Ithaca, NY, USA; ^2^Department of Biomedical Sciences, Section of Anatomic Pathology, Cornell University College of Veterinary Medicine, Ithaca, NY, USA; ^3^Department of Clinical Studies, Ontario Veterinary College, Small Animal Surgery, Guelph University, Guelph, ON, Canada

## Abstract

A 9-year-old spayed female dachshund presented with a large multilobular osteochondrosarcoma of the crania, with obliteration of approximately 70% of the surface area of the dorsal calvaria and intracranial extension. The mass was excised with histologically clean lateral bone margins (2–4 mm) and invasion at the deep margin. The resulting defect was reconstructed with a custom titanium plate. The patient recovered routinely and was asymptomatic until 7 months postoperatively. The patient developed intractable seizures 7 months postoperatively and was euthanized. Post-mortem examination showed tumor regrowth within the brain parenchyma. No abnormalities were seen associated with the plate. The patient-specific, custom additive manufactured titanium plate provided an excellent option for anatomic reconstruction and protection of the brain over a relatively large area with no complications noted.

## 1. Introduction

Various methods for pre-operative and intra-operative planning and reconstruction of extensive craniectomy have been described in dogs [1–4]. This case report describes in detail the use of computed tomographic-based 3D printing technology for creating intra-operative models and cutting guides and custom implants in this context, together with an example of deep margin soft tissue recurrence of a multilobular osteochondrosarcoma despite complete lateral margins.

## 2. Case Presentation

**Figure 1:**
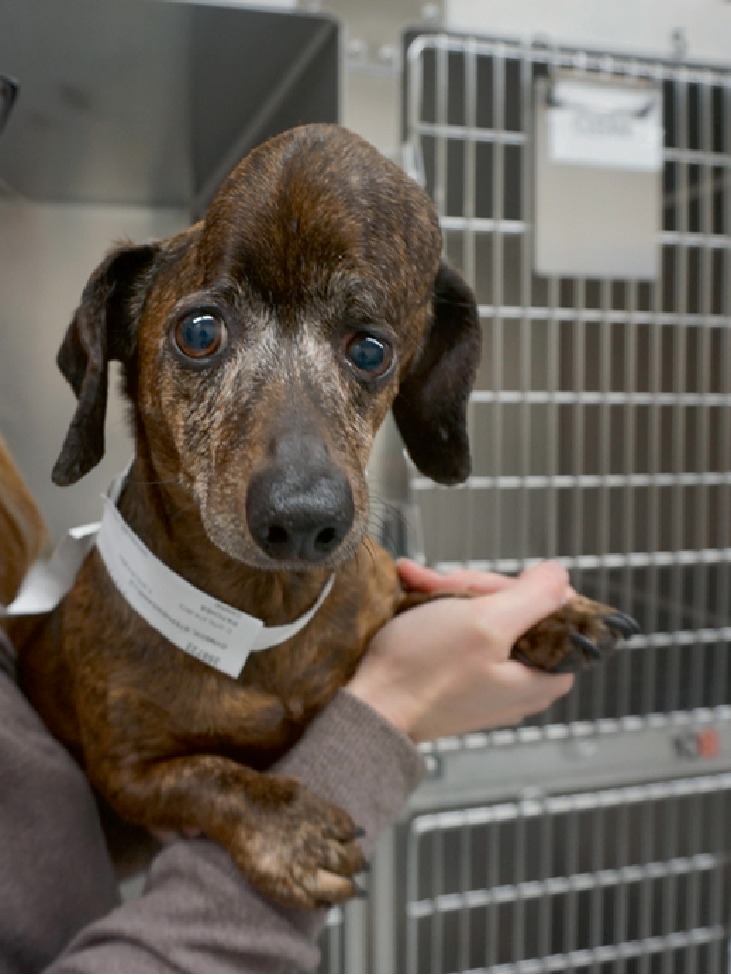
A 9 year old FS Dachshund presenting with a large multilobular osteochondrosarcoma.

A 9-year-old, 7.7 kg spayed female dachshund was referred for large tumor of the dorsal calvarium first noted by the owner 3 years prior to presentation, with a marked increase in size in the last 6 months. Tumor diameter at presentation was 7.2 × 5.2 × 6.7 cm, with an asymmetric placement toward the left side (Figure 1). A cough and serous left-sided ocular and nasal discharge was noted in the week prior to presentation. Physical and neurological exam was otherwise unremarkable. Complete blood count was unremarkable. Biochemistry profile identified a mild elevation in alkaline phosphatase at 233 U/L (reference range = 7–115 U/L). Three-view thoracic radiographs showed no abnormalities. Fine needle aspirate and cytology of the left and right mandibular lymph nodes revealed a normal to reactive small lymphocyte population and no evidence of metastasis. Under general anesthesia, a computed tomographic (CT) scan of the head was performed before and after injection of 15.4 ml of 350 mg I/ml Iohexol IV. The images were reconstructed in transverse, sagittal, and dorsal planes with four 3D surface rendered reconstructions, in bone and pre- and post-contrast soft tissue windows. The CT scan revealed a large, well-defined lobulated osseous mass arising from the left frontal and parietal bones and extending across midline. The mass consisted of irregular mineral with a classical ‘popcorn' appearance and mild heterogeneous peripheral contrast enhancement. The mass primarily extended dorsal to the calvaria with a small portion extending ventrally into the dorsal aspect of the cranial cavity causing extra-axial compression and ventro-lateral displacement of both cerebral hemispheres, worse on the left. The cerebellum showed mild caudal displacement into the foramen magnum. The mass also extended rostrally into the left frontal sinus and left orbit. The mandibular and retropharyngeal lymph nodes were normal in size. A biopsy of the mass was performed and histopathology confirmed a diagnosis of multilobular osteochondrosarcoma, also known as multilobular tumor of bone (MLO).

Surgical excision with curative-intent bone margins was recommended. To obtain these margins, resection of the roof of the left and right frontal sinuses, and a large part of the medial wall of the left orbit was required. The resulting defect necessitated cranioplasty, ideally with a rigid implant. Due to the size of the tumor and the limitations of the surrounding anatomy, the window of visualization, for performing the craniectomy through the medial wall of the left orbit, was anticipated to be small. The ability to intraoperatively view and handle a sterile anatomic bio-model of the skull and tumor for reference was anticipated to facilitate successful and accurate preoperative planning and resection.

**Figure 2:**
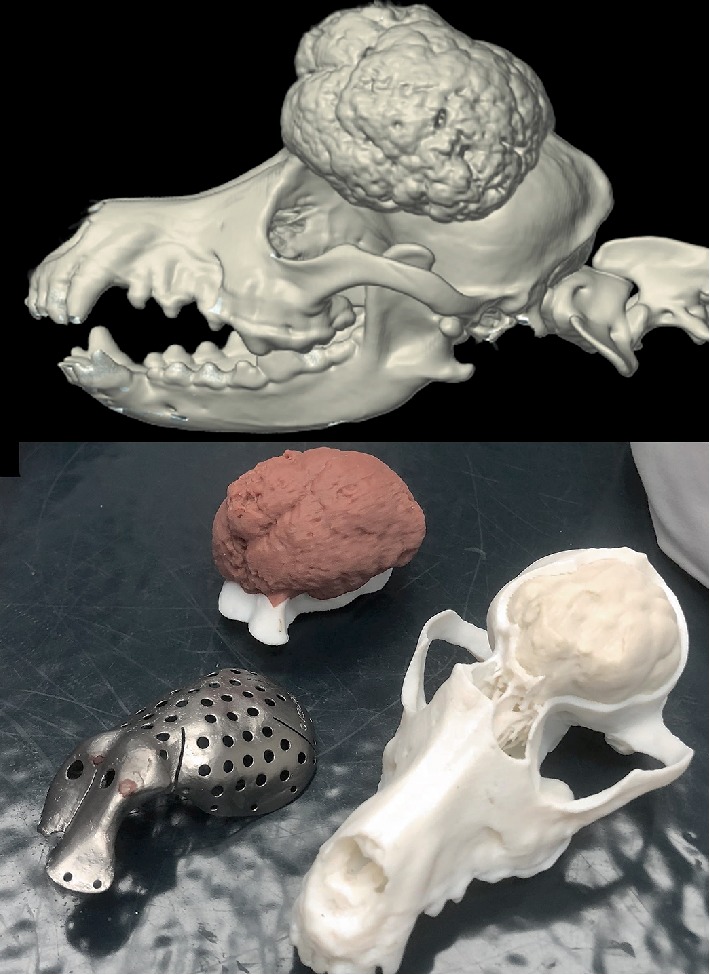
3D reconstruction of pre-operative CT and 3D printed biomodel with proposed craniectomy.

The computed tomography (CT) scans of the patient were exported as Digital Imaging and Communications in Medicine (DICOM) format and the images were imported into a DICOM viewer (Osirix MD, version 8.0.2, Pixmeo SARL, Bernex, Switzerland). A boarded veterinary surgical oncologist and radiologist assessed the scans and reached an agreement on tumor margins in Osirix MD. The DICOM files were then imported into Materialise Mimics (version 19, Materialise NV, Leuvan, Belgium) and the stereolithography (STL) file was exported using Materialise 3-matic (version 11, Materialise NV, Leuvan, Belgium). An anatomic bio-model of the patient and tumor was printed using the J750 polyjet 3D printer (Figure 2).

A custom titanium plate was designed and printed for the patient as described by James et al. [5]. Briefly, a digital defect was created from the previously identified tumor margin with a 5 mm safety margin from the defined tumor boundaries and the file exported. Utilizing medical 3D printing service hub, Additive Design in Surgical Solutions (ADEISS, London, ON), the plate was designed using ADEPT (version 2017, Renishaw PLC, Wotton-under-Edge, Gloucestershire, United Kingdom) cranial plate creation software. The plate was printed in titanium and processed as previously described.

A cutting guide was then created to provide a template for surgical resection. First, the tumor margins identified in Osirix MD were imported into ANSYS SpaceClaim (version 2017, ANSYS incorporated, Canonsburg, Pennsylvania, United States). A 3D spline curve was fit to the data points which represented the tumor margin. The reconstructed skull STL and the tumor margin curve were imported into Geomagic Freeform (version 2017, 3D systems incorporated, Rock Hill, South Carolina, United States). The guide was designed to fit to the interface between the skull and tumor surfaces with a positive profile to be used for the template. The tumor margin curve was thickened into a 10 mm diameter 3D round tube and subtracted from the skull creating a model of the skull with a 5 mm margin tumor resection. The cutting guide was created by subtracting the intact skull STL from the 10 mm 3D round bar. The resulting shape was divided into four sections. The cutting guide segments were printed for intraoperative use.

The STL models of the cut skull, 5 mm margin tumor resection, tumor, and surgical cutting guides were 3D printed using the J750 polyjet printer (Stratasys, Eden Prairie, Minnesota, United States). The titanium plate was autoclave sterilized under standard conditions. The guide and skull pre- and post-planned craniectomy, were H2O2 sterilized with standard protocols for use in the operating room.

**Figure 3:**
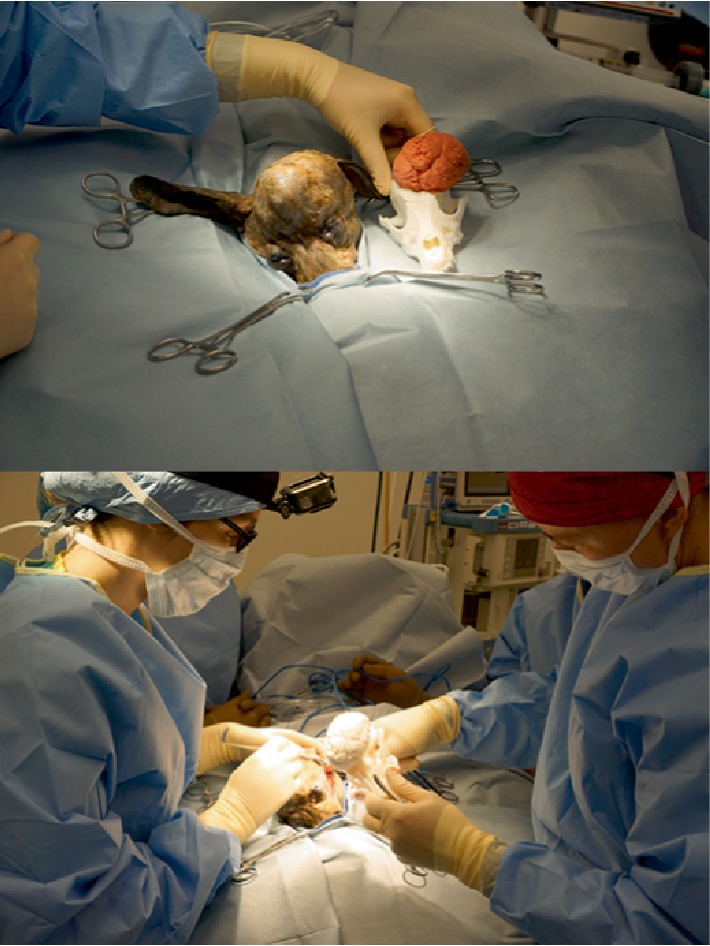
Biomodel in use intra-operatively for planning orbitectomy site.

**Figure 4:**
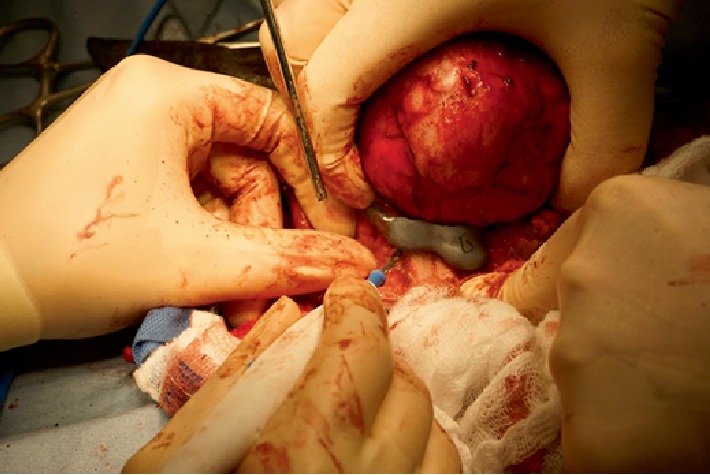
3D printed cutting guide in use to mark resection margin.

**Figure 5:**
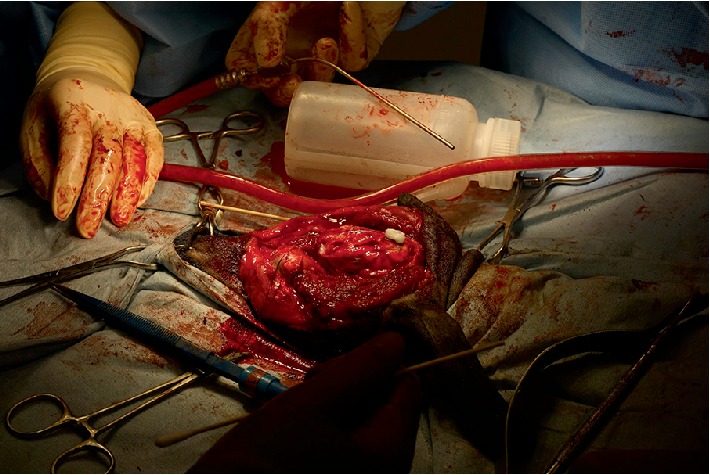
Extent of brain exposure following craniectomy. Stay suture marks descended left eye following zygomatic arch resection and orbitectomy. The medial wall of the orbit was resected to within 5 mm of the optic nerve foramen on the left side.

**Figure 6:**
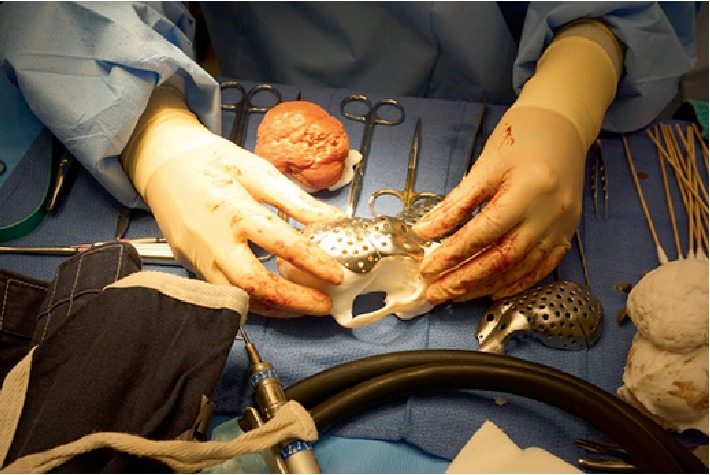
Plate contouring was re-checked against the 3D model and patient.

**Figure 7:**
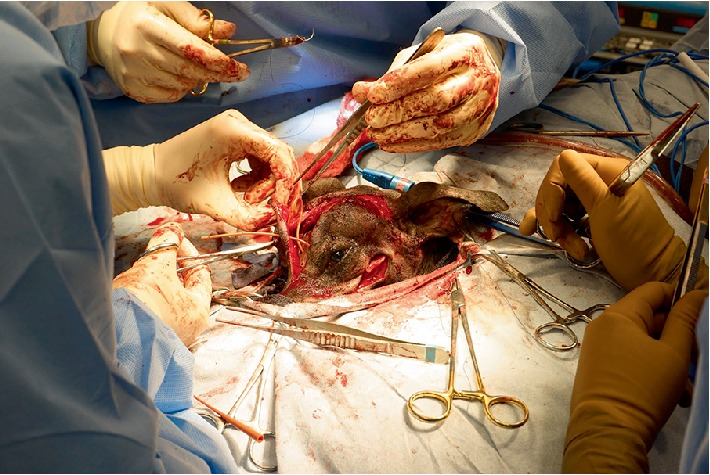
Intra-operative view following reconstruction and restoration of normal eye position.

Following construction, printing and sterilization of the implant, the patient was admitted for surgery. The patient was pre-medicated with dexmedetomidine and atropine. Anesthesia was induced with remifentanil and propofol and maintained with remifentanil and propofol constant rate infusion (CRI). Neuromuscular blockade was initiated with an atracurium CRI and positive pressure ventilation on 100% oxygen provided. Cefazolin was administered prior to skin incision. Phenylephrine was administered to control blood pressure. Continuous monitoring of EKG, SpO2, esophageal temperature, end tidal CO_2_ and level of neuromuscular blockade (train of four) was performed. Surgical excision of the mass was performed as per a standard craniectomy approach with modifications to allow for access to the ventral orbit [6]. Briefly, the patient was positioned in sternal recumbency with the head supported on a vacuum positioner (DRE veterinary, Louisville, KY) in a manner to minimize pressure on the jugular veins. A skin incision was made along the dorsal margin of the left zygomatic arch and extended to the level of the bone. With reference to the 3D model, osteotomy of the zygomatic arch was performed to improve orbital access (Figure 3). The orbital ligament was transected close to the insertion on the zygomatic process and the peri-orbital fascia along the dorsal margin of the upper palpebral muscle was incised. This allowed reflection of the zygomatic arch and globe ventrally giving access to the dorsal retrobulbar space and medial wall of the orbit. A second skin incision was then made along the dorsal midline of the skull from a point level with the medial canthi to the level of the occiput. The skin incision was extended with 1 cm margins around the previous biopsy tract for excision. Once the tumor margins were exposed, the cutting guide was placed around the tumor and electrocoagulation used to mark the excision margin (Figure 4). A 3–5 mm Hall's air burr was used to perform the craniectomy. Hemostasis was achieved with bone wax and penetration of the dura was avoided. A small defect in the sagittal venous sinus was repaired with 6/0 gortex suture and gel foam. Following completion of the craniectomy line, periosteal elevators were used to apply dorsal elevation to the segment and fracture the attachment of the cribriform plate (Figure 5). Following exposure of the brain surface a small tear in the dura was noted and patched by apposition of a sheet of porcine SIS. Additional SIS was applied to the remainder of the exposed area, with a double layer in the area adjacent to the frontal sinuses. Contouring was re-checked (Figure 6) and the pre-constructed titanium plate was placed over the defect and secured with four 1.5 mm titanium self-tapping screws. A partial thickness rotational flap was created from the temporalis musculature and used to achieve soft tissue coverage of the majority of the caudal aspect of the implant. Two 5Fr red rubber tubes were placed through two pre-planned holes in the implant that provided access to the frontal sinuses and exited through the skin to prevent air entrapment in the early postoperative period (Figure 7). The zygomatic arch was repaired and all tissue layers apposed routinely.

**Figure 8:**
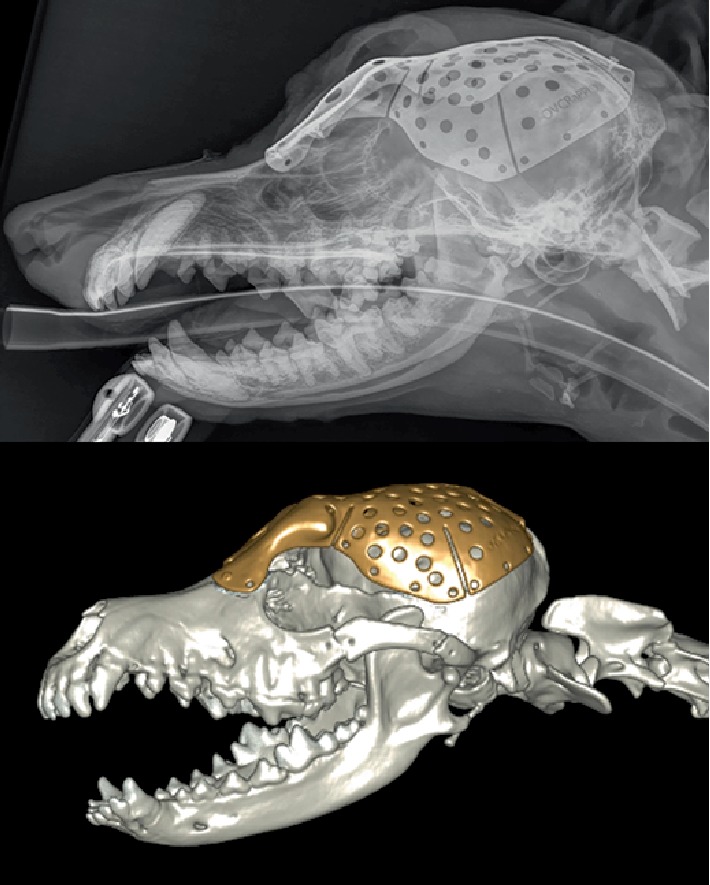
Post-operative CT scan.

The patient made a routine recovery from surgery and was maintained postoperatively on intravenous fluid therapy, fentanyl (1–6 ug/kg/hr IV CRI) and cefazolin (22 mg/kg IV Q8). Fluorescein staining of the left eye the evening of surgery was negative for injury, and palpebral, menace and pupillary light reflexes were normal. The dog was neurologically appropriate following anesthesia recovery and was weaned off of analgesics over 2 days postoperatively and discharged 3 days postoperatively on gabapentin 50 mg PO Q12 and clavaseptin 62.5 mg PO Q 12 × 14 days. The tubes in the frontal sinuses were removed prior to discharge and the stomas allowed to granulate by second intention healing. No subcutaneous emphysema was noted at any point. Mild serosanguinous nasal discharge was noted for the first 5 days postoperatively which then resolved. Incisional healing was routine. A CT scan was repeated under sedation 14 days postoperatively, and showed good skull defect coverage and alignment of the implant (Figure 8). Histopathology of the excised tissue confirmed diagnosis of a multilobular osteochondrosarcoma with clean lateral margins ranging between 2 and 5 mm. Extension of the tumor into the calvaria was noted on the deep margin, but was presumed to constitute disease extension rather than invasion. The patient experienced intervertebral disc herniation and loss of pelvic limb nociception 14 days postoperatively. Routine decompressive L2/3 hemilaminectomy was performed but nociception or ambulation were never regained.

The patient continued to have no evidence of clinical signs associated with the craniectomy or cranial plate until 6 months postoperatively. At that time, focal seizures were noted. Advanced imaging was declined and treatment with phenobarbital 5 mg/kg resulted in control of seizures until 7 months postoperatively when the patient experienced 4 seizures over 3 days and was presented in status epilepticus. Humane euthanasia was elected due to a progression of clinical signs and an autopsy was performed.

**Figure 9:**
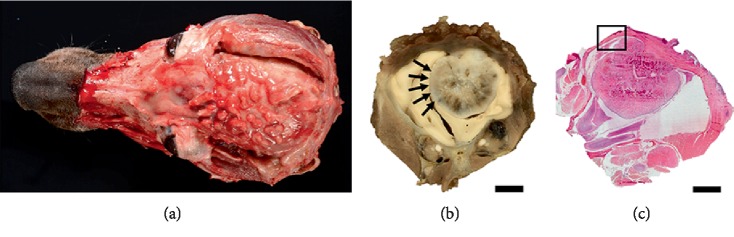
Multilobular osteochondrosarcoma regrowth after excision and calvaria replacement with titanium implant. (a) Gross aspect of the brain-plate interface after post-mortem removal of the plate. Note the thin fibrous tissue that was extending through the plate's holes. (b) Section through the entire skull at the level of the frontal cortex, post fixation and decalcification. Note the well-demarcated, intraparenchymal compressive mass (black arrows) with small white foci of bone in the structure, compatible with multilobular osteochondrosarcoma (bar = 1 cm). (c) Subgross photomicrograph of the previous section (b). Note the intraparenchymal growth of the mass, attached to the fibrous tissue starting under the fibrovascular tissue (bar = 1 cm; Hematoxylin and eosin (H&E) stain).

**Figure 10:**
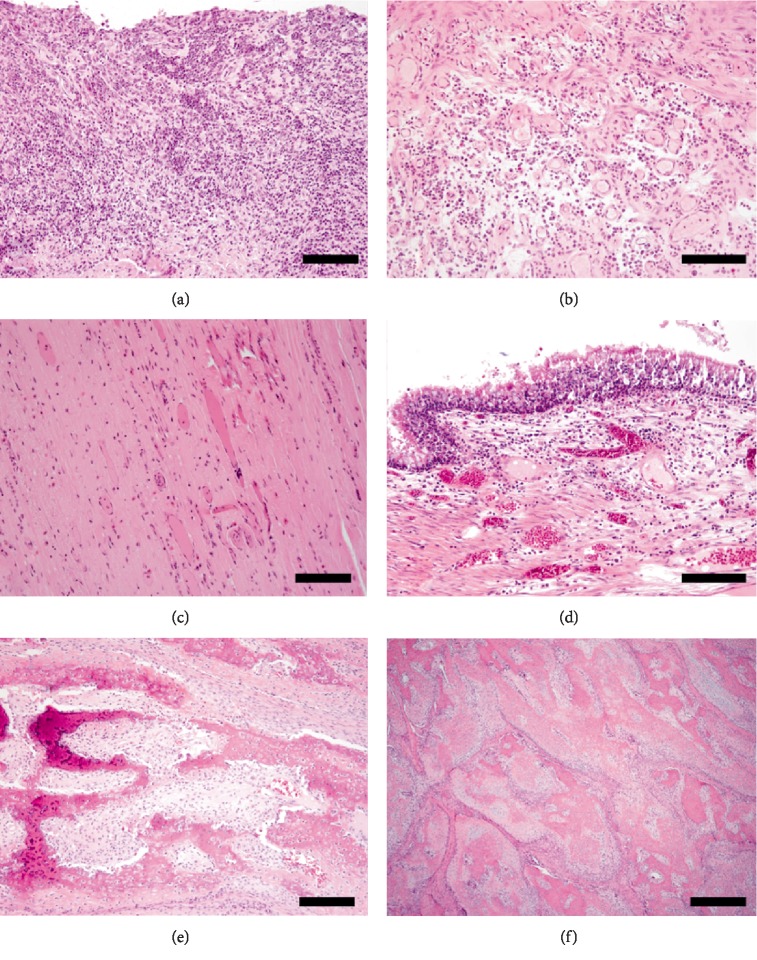
(a) Inflamed granulation tissue immediately under the titanium plate. Note the moderate, predominantly neutrophilic inflammation (bar = 300 *µ*m; H&E stain). (b) Tapering of the inflammation towards the porcine SIS with lymphoplasmacytic predominance and abundant small caliber blood vessels supporting neovascularization (bar = 300 *µ*m; H&E stain). (c) Porcine SIS with appropriate vascularization and no evidence of inflammation immediately covering the brain (bar = 300 *µ*m; H&E stain). (d) Photomicrograph of sinus epithelium revealing a mild to moderate neutrophilic and lymphoplasmacytic sinusitis (bar = 250 *µ*m; H&E stain). (e) Photomicrograph of the multilobular osteochondrosarcoma at the time of surgical excision, revealing lobules of thick tendrils of spindle cells that are enclosing plump polygonal cells admixed with abundant extracellular eosinophilic matrix, interpreted as osteoid, findings typical of this neoplasm (bar = 250 *µ*m; H&E stain). (f) Photomicrograph of the multilobular osteochondrosarcoma regrowth revealing similar organization as previously noted (Figure 10(e)) with slightly less cellular atypia and more organized lobules (bar = 250 *µ*m; H&E stain).

Grossly, the titanium plate was well-placed, surrounded by a granulation tissue bed with granulation tissue filling the holes of the titanium plate. Suppurative sinusitis (Figure 10(d)) that cultured beta-hemolytic E. coli affected the frontal sinuses that tracked along the fascia around the titanium plate (Figures 9(a) and 10(a)). Histologically, this inflammation attenuated into a lymphoplasmacytic infiltrate towards the porcine SIS (Figure 10(b)); but the porcine SIS was mostly uninflamed. Neovascularization was noted within the porcine SIS (Figure 10(c)). The lack of inflammation of the porcine SIS and presence of neovascularization suggested that the porcine SIS was well accepted by the host. The sinus inflammation minimally penetrated into the meninges of the olfactory lobe but did not affect the brain parenchyma. Cross section of the brain revealed a 1.8 cm intracerebral mass (Figures 9(b) and 9(c)) replacing most of the right frontal cortex, causing localized tissue damage, and significant midline shift. Histologically, compared to the previous excision (Figure 10(e)), the intracranial regrowth had less cellular atypia and larger, coalescing lobules (Figure 10(f)). Histologically, both were classified as grade II based on the grading scheme [7].

## 3. Discussion

This report demonstrates the successful use of a custom additive manufactured titanium plate in a dog. Several implants are available for reconstruction following craniectomy [1–4]. Titanium mesh is deformable and may not provide an adequate physical barrier in the context of a large area craniectomy. Polymethyl methacrylate is inexpensive and readily available, but may be challenging to (1) secure over a large area, (2) requires making an intraoperative mold which extends surgical times, and (3) has been associated with increased risk of implant infection particularly when exposed to a bacterial population from the frontal sinuses [8]. While custom printed titanium implants are available for human reconstructive surgery, we are not aware of a previous report of this implant type for craniectomy reconstruction in the dog. The rigidity of the implant, relative resistance to infection, ability to construct the implant entirely preoperatively with no intraoperative delays necessary, and the extreme customizability of the implant to the wide variety of skull shapes and sizes found in the canine, may be advantages over other implants.

In addition to a custom plate, 3D printing was utilized in this case to create an anatomic bio-model of the patient and a cutting template. The intraoperative availability of a sterile, to-scale model of the tumor and proposed craniectomy provided several advantages [9]. For this case, the window of visualization for the segment of the craniectomy that formed the medial wall of the left orbit was relatively limited—despite release of the zygomatic arch, a long burr guard was needed to bypass the surrounding soft tissues, and the bone was relatively thin in this area. The bio-model allowed the surgeon to orientate more accurately to precise location on the bone despite the soft tissue coverage. It also provided real time information on the thickness of the bone in the region undergoing burring, which allowed burring to proceed faster. In addition, having a model for verbal orientation and co-ordination of the rest of the surgical team was helpful, to allow for referencing the model, improving intraoperative communication between team members.

Since the procedure was performed in a single-stage it was important that the resultant craniectomy defect was precise to ensure adequate margins for aspects of the mass that were not visible on the surface and to ensure the implant would fit for reconstruction. Cutting guides, or templates, have been described for a number of surgical procedures in both human and veterinary surgery [10–14]. Templates are designed to assist the surgeon in where to place the incisions to improve precision of the procedure. In this case, the guide was created by identifying the tumor margins on the CT and adding an additional safety margin beyond the tumor. One of the limitations of the template is that it often needs to be divided into several parts to accommodate the shape of the tumor. To accomplish this, each of the parts needs to conform to specific landmarks to ensure placement accuracy. As there is a period of time between the planning and procedure, the guide cannot conform too closely to the tumour, as the tumor may have changed slightly during the planning process. Our simplistic guide allowed us to trace the planning resection margin and act as a means to ensure an adequate margin while still achieving plate coverage. In the future, this guide could be adapted to aid guiding the depth of the skull in the various areas.

Any use of surgical implants carries a lifelong risk of implant-associated infections. In this case, the craniectomy involved removal of both a large portion of the frontal sinus and calvarium, including the cribriform plate, resulting in a communication between the sinonasal and calvarial cavity. Reconstruction of this division was not possible and porcine SIS was placed to provide a pseudo-barrier and to decrease the risk of adhesions. Antimicrobial prophylaxis was provided for 2 weeks and no evidence of clinical infections were noted. At the time of post-mortem examination, approximately 7 months postoperatively, there were signs of sinusitis but there was no indication of any active intracranial infection. In humans, infection in patients with cranial implants has been reported in 7–16% [15–17]. It is suspected that with increasing number of cases, this figure will be similar for veterinary patients.

In this case, the patient developed status epilepticus and was euthanized 7 months postoperatively. Initially the differential diagnoses for seizures included infection, adhesions or hydrocephalus. Given the presence of a complete histologic lateral bone margins and no evidence of intradural invasion at the time of the initial surgery, recurrence of the tumor within the dural tissue was not expected. Absence of a deep margin on the original histopathology report was interpreted as disease extension into the calvaria rather than infiltration of the intra-calvarial soft tissues. However, based on the autopsy findings, it was determined that the cause of the seizures was the presence of a large, intracerebral mass compressing and replacing most of the right frontal cortex, causing significant midline shift. The tumor appeared to be extending from the fibrovascular tissue abutting the brain and was contained beneath the porcine SIS and growing into the brain tissue. The histologic features of the regrowth compared to the initial surgical excision (Figure 10(e)) have slight differences. The size of lobules are larger and broader with frequent coalescing trabeculae and the lobules are poorly organized (Figure 10(f)). Collectively, both the excised tumor and current tumor are considered grade II out of III [7]. Incomplete or even narrow excision is thought to lead to more rapid regrowth. The ingrowth into the cerebrum rather than out invading the porcine xenograft may reflect the path of least resistance but it may also reflect the microenvironment; the cerebrum is normally vascularized compared to the porcine that needed to be vascularized. These findings were unexpected and demonstrate a potential need to consider the dura mater as a deep margin in cases of MLO in dogs. In the veterinary literature, recurrence rates for MLOs are high, with 47% of cases developing local recurrence in one study. In that study, completeness of bone resection margins was considered prognostic for recurrence and 41% of tumors were resected with incomplete margins. The study was not limited to calvarial MLOs and the tissue of origin for recurrence was not stated [7]. A similar case with rapid regrowth was reported [18]. In the cited case, the authors commented on the possibility of the entire procedure, along with medication, leading to an environment conducive for regrowth of remaining neoplastic cells. Among factors discussed are hypoxia within malignant tissue, tumor-derived immunosuppression, inflammatory environment, and immunosuppressive doses of dexamethasone. Generally, surgical excision, even with incomplete margins, with or without additional therapy, is expected to offer a long tumor-free interval, with a median of 22 months [19] that differs from the current case.

To the authors' knowledge, this is the first report of recurrence of MLO in the soft tissues surrounding the brain.

This case report demonstrates the successful use of 3D printing for creation of a surgical cutting guide and custom additive manufactured titanium plate in a dog following single-stage craniectomy and cranioplasty. Based on our experience, this technique appears feasible with excellent cosmesis and limited postoperative complications. Local recurrence should be considered, as with any case of tumor resection, and soft tissue margins may be more important than previously reported in cases of MLO in dogs.
